# Development and Validation of an Artificial Intelligence System to Optimize Clinician Review of Patient Records

**DOI:** 10.1001/jamanetworkopen.2021.17391

**Published:** 2021-07-23

**Authors:** Ethan Andrew Chi, Gordon Chi, Cheuk To Tsui, Yan Jiang, Karolin Jarr, Chiraag V. Kulkarni, Michael Zhang, Jin Long, Andrew Y. Ng, Pranav Rajpurkar, Sidhartha R. Sinha

**Affiliations:** 1 Department of Computer Science, Stanford University, Stanford, California; 2 Division of Gastroenterology and Hepatology, Department of Medicine, Stanford University, Stanford, California; 3 Department of Neurosurgery, Stanford University, Stanford, California; 4 Center for Artificial Intelligence in Medicine and Imaging, Stanford University, Stanford, California

## Abstract

**Importance:**

Physicians are required to work with rapidly growing amounts of medical data. Approximately 62% of time per patient is devoted to reviewing electronic health records (EHRs), with clinical data review being the most time-consuming portion.

**Objective:**

To determine whether an artificial intelligence (AI) system developed to organize and display new patient referral records would improve a clinician’s ability to extract patient information compared with the current standard of care.

**Design, Setting, and Participants:**

In this prognostic study, an AI system was created to organize patient records and improve data retrieval. To evaluate the system on time and accuracy, a nonblinded, prospective study was conducted at a single academic medical center. Recruitment emails were sent to all physicians in the gastroenterology division, and 12 clinicians agreed to participate. Each of the clinicians participating in the study received 2 referral records: 1 AI-optimized patient record and 1 standard (non–AI-optimized) patient record. For each record, clinicians were asked 22 questions requiring them to search the assigned record for clinically relevant information. Clinicians reviewed records from June 1 to August 30, 2020.

**Main Outcomes and Measures:**

The time required to answer each question, along with accuracy, was measured for both records, with and without AI optimization. Participants were asked to assess overall satisfaction with the AI system, their preferred review method (AI-optimized vs standard), and other topics to assess clinical utility.

**Results:**

Twelve gastroenterology physicians/fellows completed the study. Compared with standard (non–AI-optimized) patient record review, the AI system saved first-time physician users 18% of the time used to answer the clinical questions (10.5 [95% CI, 8.5-12.6] vs 12.8 [95% CI, 9.4-16.2] minutes; *P* = .02). There was no significant decrease in accuracy when physicians retrieved important patient information (83.7% [95% CI, 79.3%-88.2%] with the AI-optimized vs 86.0% [95% CI, 81.8%-90.2%] without the AI-optimized record; *P* = .81). Survey responses from physicians were generally positive across all questions. Eleven of 12 physicians (92%) preferred the AI-optimized record review to standard review. Despite a learning curve pointed out by respondents, 11 of 12 physicians believed that the technology would save them time to assess new patient records and were interested in using this technology in their clinic.

**Conclusions and Relevance:**

In this prognostic study, an AI system helped physicians extract relevant patient information in a shorter time while maintaining high accuracy. This finding is particularly germane to the ever-increasing amounts of medical data and increased stressors on clinicians. Increased user familiarity with the AI system, along with further enhancements in the system itself, hold promise to further improve physician data extraction from large quantities of patient health records.

## Key Points

**Question:**

Would the development of a novel artificial intelligence (AI) system to organize patient health records improve a physician’s ability to extract patient information?

**Findings:**

This prognostic study of 12 physicians or fellows in an academic gastroenterology department found that first-time physician users of the AI system were able to save a mean of 18% of the time taken to answer clinical questions regarding a patient’s medical history while maintaining accuracy comparable to their performance without AI.

**Meaning:**

These findings suggest that, without sacrificing accuracy, the AI technology developed helps physicians extract relevant patient information in a shorter time.

## Teaser

This prognostic study evaluates the utility of and potential time savings, accuracy, and user experience associated with an artificial intelligence–optimized electronic health record tested by 12 gastroenterologists.

## Introduction

It has been estimated that more than one-half of a general clinician’s workday can be spent interacting with electronic health records (EHRs).^1,2,3,4,5^ A survey of more than 500 primary care physicians^6^ reported that 62% of time devoted to each patient visit is spent referring to EHRs. Recently, a large study of approximately 100 million patient encounters across multiple medical specialties concluded that medical record review (ie, reviewing clinical results, patient data, and notes) accounted for the largest segment of time spent in the EHR, a trend also seen in inpatient medicine.^7,8,9^ Along with the increased adoption of EHRs, there has been a concomitant increase in the amount of data stored in these systems.^10^ Consequently, a theme of challenges experienced by EHR users is information overload, particularly because much of the EHR data (including new patient referral information) is often not pertinent to the particular patient encounter.^11,12,13,14^ Many factors—including overwhelming amounts of data—associated with EHR use have been contributors to physician dissatisfaction and burnout.^15,16,17,18^

In referral-based medical specialties, clinicians receive patient records containing medical histories that can range in size from several to hundreds of pages, depending on complexity. The information contained in these records—including clinical notes, laboratory values, radiology reports, procedure notes, and pathology findings—is crucial to providing a sound consultation. In most instances, faxed records from referring clinicians are scanned into the EHR before being reviewed by the consulting physician. Owing to issues such as the presence of extraneous information, redundant notes, lack of a search ability, poor organization, and unstandardized formats, this review process can be time-consuming and prone to error, ultimately reducing the amount of time spent directly with patients and potentially increasing duplicative and costly orders.

In this study, we describe the development and testing of an artificial intelligence (AI) system designed to increase efficiency of reviewing and extracting clinically relevant data from patient referral records. Although its incorporation into routine practice has not been widespread, the use of AI systems has been explored in a wide variety of clinical scenarios across multiple medical specialties.^19,20,21,22,23,24^ To our knowledge, it has yet to be applied to the review of referral records, a process common to all medical specialties. Our AI system extracts and organizes relevant patient information and presents it to physicians alongside the entire scanned medical record in a web-based user interface. To evaluate the utility and potential time savings during medical record review, physicians were asked to answer clinically relevant questions after review of medical records using our software vs standard (ie, non–AI-optimized) records. In addition to time savings, we assessed secondary measures, including accuracy and user experience.

## Methods

### Ethical Review

This prognostic study was approved by the Stanford University institutional review board, and all participants provided written informed consent. This study reported on items in the Standards for Reporting of Diagnostic Accuracy (STARD) and Transparent Reporting of a Multivariable Prediction Model for Individual Prognosis or Diagnosis (TRIPOD) reporting guidelines.

### Data Acquisition

**Figure 1.**
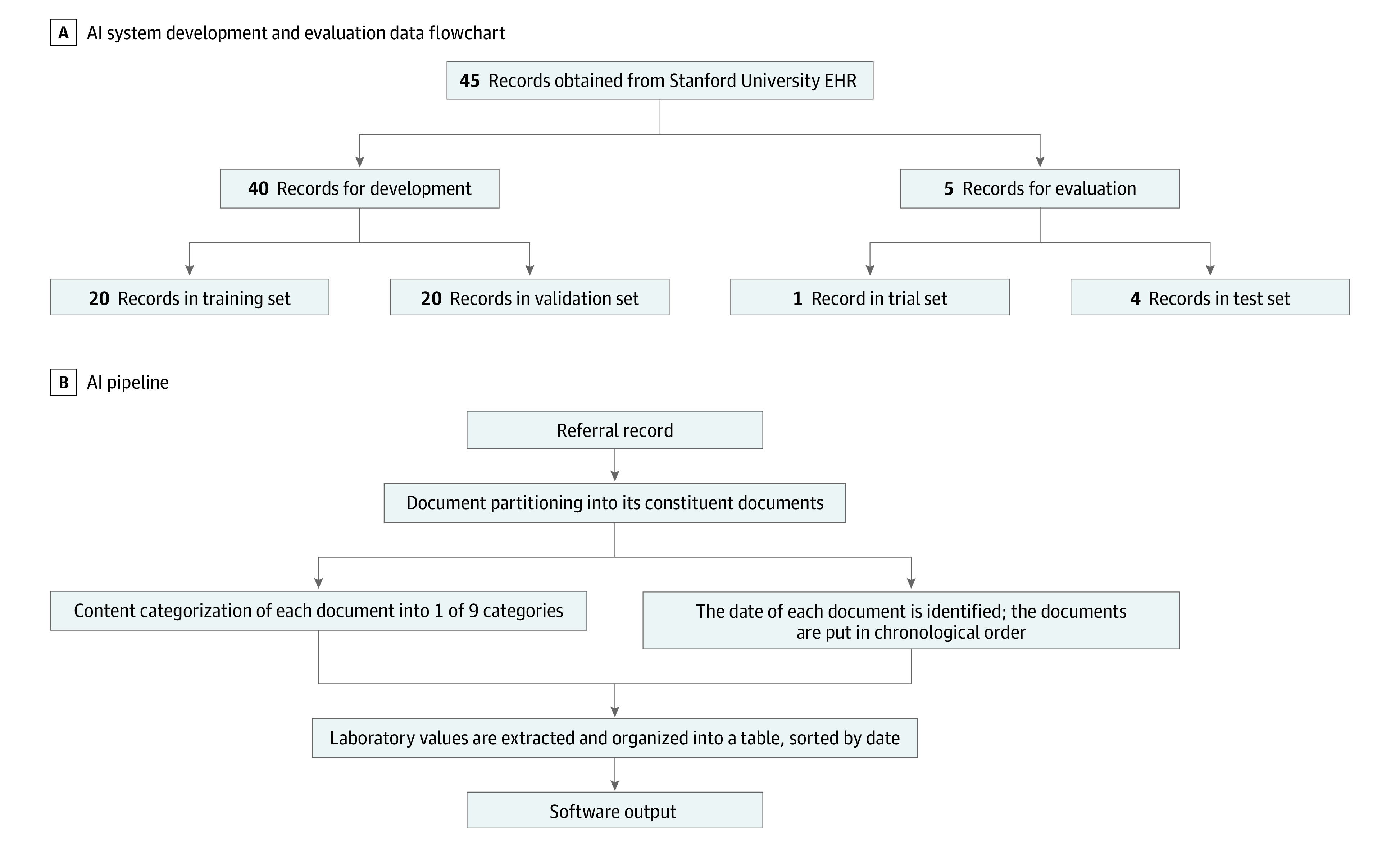
Data Flowchart and Artificial Intelligence (AI) System Pipeline A, Forty-five readable patient referral records from gastroenterology clinicians were randomly obtained for the development of the system. They were separated into training, validation, and test sets. Three of the training set scans were manually annotated to identify information categories for the system to extract. Five records were used for physician evaluation: 4 for testing and 1 for collecting initial user feedback. B, The input is a scanned referral record, which is first partitioned into its constituent documents. The document is then classified into 1 of 9 categories (or undetermined): referral, note, laboratory, radiology, procedure, operative report, pathology, fax cover sheet, or insurance. The documents are ordered by their most recent date. Laboratory values are extracted and presented in a table, sorted by date. EHR indicates electronic health record.

Our system was developed using 60 patient referral records from the Division of Gastroenterology and Hepatology at Stanford University, Stanford, California. New clinic patient referral records were chosen from a variety of gastroenterology clinicians at Stanford University, including multiple subspecialities (eg, liver disease, motility, inflammatory bowel disease). The records were selected at random from our institution’s EHR, with 15 records removed owing to poor scanning quality. Of the remaining records, the training and validation sets both included 20 records. Three of the training set records were thoroughly annotated with clinician input for the purpose of identifying the types of relevant information for the AI system to extract. An independent test set of 4 records was used for evaluation; these records were chosen randomly but were required to be sufficiently well scanned so that the text could be recognized, and the record was required to contain at least 1 laboratory table, 1 progress note, and 1 of the following: procedural report, radiology report, operative report, or pathology report (Figure 1A). Of the 5 records used for evaluation, 1 record was used as a trial run to collect initial user feedback from a physician; the remaining 4 records were used for testing.

### Development of the AI System

The system comprised a pipeline of AI algorithms to organize relevant clinical information from a patient referral record and present information to the clinician in a web interface. The pipeline of AI algorithms consisted of algorithms to (1) read text in PDF to extract dates, laboratory findings, and social history and (2) organize the record’s pages by content category (referral, fax, insurance, progress note, procedure note, radiology report, laboratory values, operative report, or pathology report) (Figure 1B). Technical details of the pipeline, which was developed from September 2019 to May 2020, are described in the eMethods in the Supplement.

**Figure 2.**
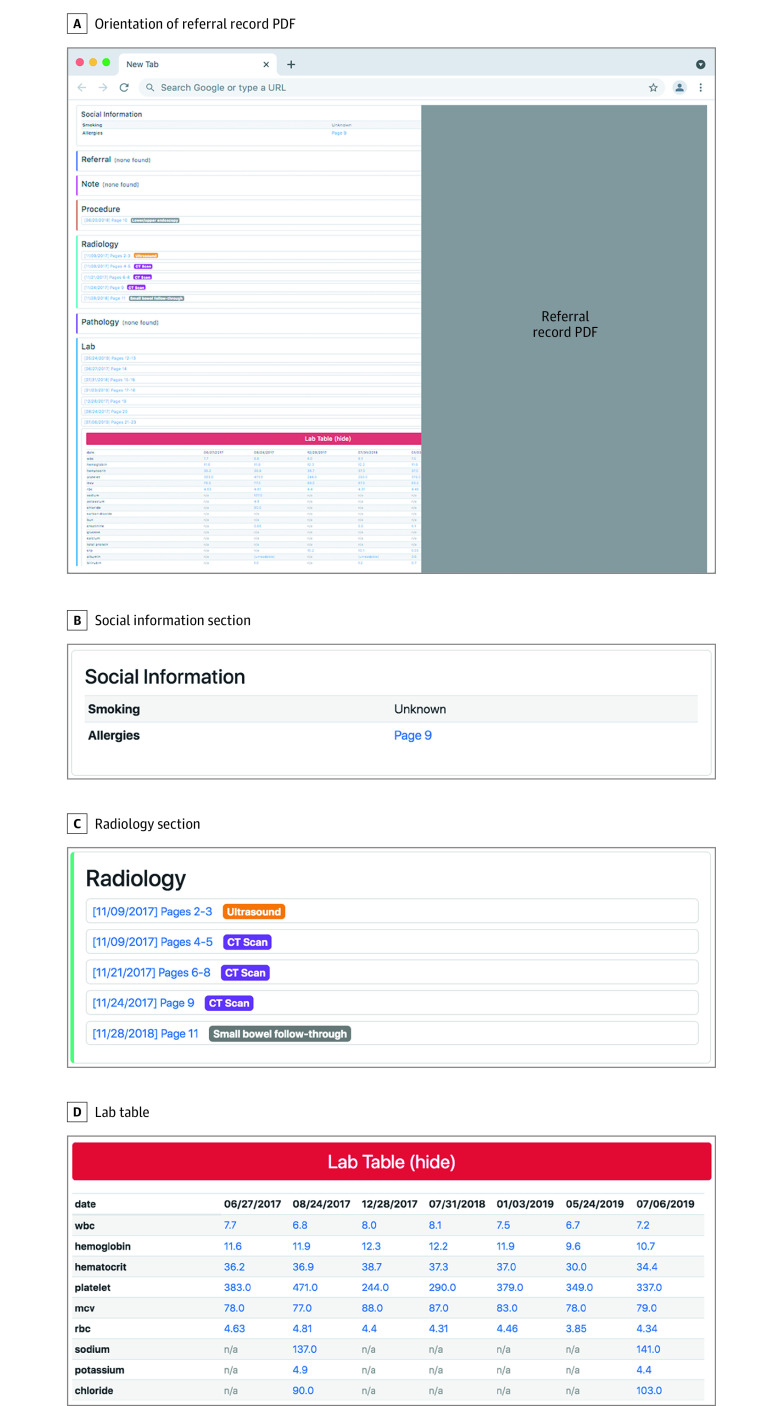
User Interface With Artificial Intelligence (AI)–Optimized Record A, The original referral record PDF is displayed on the right of the interface. The AI output is shown on the left in 3 representative sections: B, the social information section, which contains smoking and allergy information; C, the radiology section, which displays items that are predicted to belong to the radiology category; and D, the laboratory table, which organizes by date the laboratory values extracted from the document. In practice, 1 section for each category would be predicted by the system to be in the document.

We first developed a date extraction algorithm to discern the dates from a group of pages in the record. Subsequently, we created an algorithm to identify laboratory values in the record and organized the results in a distinct table. A content categorization model was developed to organize the record by the following categories: referral, note, laboratory, radiology, procedure, operative report, pathology, fax cover sheet, or insurance (eTable 1 in the Supplement). Finally, a page-grouping algorithm, using a convolutional neural network and textual heuristics, was developed to partition the record into its constituent documents. To present the optimized patient information to the clinician, we developed a web interface that displayed the outputs of the system for a given referral record. Displayed on the left side of the interface was a summary containing a list of document categories found in the record, along with hyperlinks to the original full PDF record, which was shown on the right side of the interface in its entirety (Figure 2). All the information in the original referral was put through these algorithms and categorized by the AI system.

### Study Design

**Figure 3.**
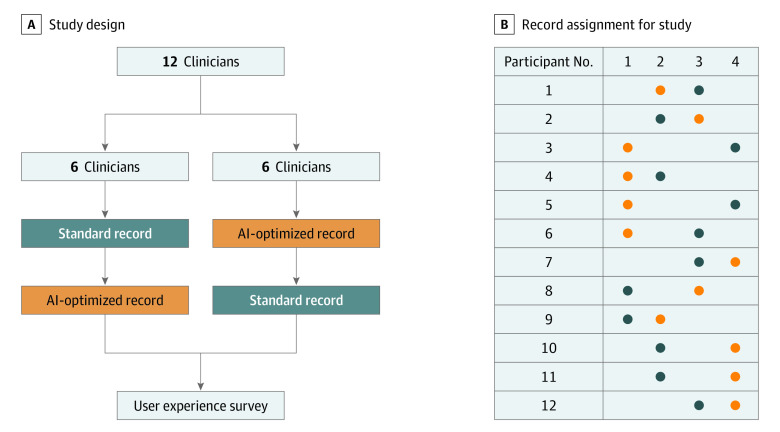
Study Design and Record Assignment for Study A, Clinicians were assigned 1 standard and 1 artificial intelligence (AI)–optimized record in random order. B, Combination of records assigned to each participant.

The goal of this study was to evaluate whether our system would reduce the time needed to extract clinical information from patient referral records. In addition, we sought to assess the accuracy of the extracted data. A total of 12 clinicians from Stanford School of Medicine’s Division of Gastroenterology and Hepatology were recruited after consenting to the study. In our study conducted from June 2020 to August 2020, each of the clinicians taking part in the study received 2 referral records to review: 1 AI-optimized record and 1 standard referral record (Figure 3). Records were randomly assigned to the AI-optimized vs standard review on a per-subject basis such that each record was seen approximately an equal number of times. Record order was also randomized across clinicians to avoid confounding by reader fatigue.

For each record, clinicians were asked to access a web-based testing interface designed to assess the AI system, which integrated a built-in training session. First, clinicians read a set of instructions that explained the goal of the study and the study setup, including the set of clinical questions they would need to answer. Second, they watched a brief training video that highlighted the features of the system and demonstrated how to navigate the web interface. Third, they were asked to answer 3 example questions on a demonstration record. The workflow was set up such that participants had to complete each step before moving to the next step of the process.

After completing the brief training, clinicians were asked to answer 22 clinical questions that required them to search and extract information from their assigned record (eTable 2 in the Supplement). There were 19 overall questions, with 3 of those 19 questions requiring 2 answers (eg, identifying a laboratory value and the date that laboratory value was obtained), resulting in 22 discrete questions total for each referral record. The time to answer each question and the accuracy of each answer was recorded. The questions were developed with gastroenterologists, who did not have direct access to coding of the algorithm, to simulate the workflow of clinicians when reviewing patient records by testing data extraction from sections of a patient referral record commonly reviewed, including progress notes, laboratory data, radiology reports, and medication lists. Only 1 question was shown at a time; a displayed timer started as soon as the question appeared on the web interface and ended only after the question was answered and submitted. Each clinician completed this full set of questions twice: once for a standard patient record and once for an AI-optimized patient record.

After answering the questions for both assigned records, clinicians completed a user experience survey that asked them to rate the software on measures of ease of use and convenience (eTable 3 in the Supplement). The survey was designed to assess the overall physician experience and opinions on clinical utility of the AI system. It included both standard metrics such as likelihood to recommend as well as questions addressing limitations that can be used to improve on future iterations of the system. The results of these questions were used to report clinicians’ overall satisfaction with the software, as well as whether the software was effective enough for potential use in a clinical setting.

### Statistical Analysis

The time taken to answer standardized clinical questions by the participants with and without AI optimization was assessed using a linear mixed-effects model. For this model, the parameter of interest was the fixed-effects indicator for whether the question was read with AI optimization or not. A random effect was included to account for the variability across both the participants and questions. In addition, we included the accuracy and record size as covariates in the model for the potential confounding effects. Similarly, the accuracy of the clinicians with and without AI optimization was compared using a logistic mixed-effects model with similar settings on the fixed and random effects. To evaluate the effect of AI optimization accounting for the record size, the time difference between AI optimization and standard review were estimated from the mixed-effects model for each participant. To evaluate the benefit from our software, a correlation matrix was analyzed to estimate the time saved by AI optimization for each physician. To explore the association between standard record review time and time saved by using the AI system, a Pearson correlation coefficient was calculated. All models and statistical analyses used SAS, version 9.4 (SAS Institute, Inc). Statistical significance was set at *P* = .05, and all tests were 2-tailed.

In addition, date extraction and page classification were evaluated on accuracy, and accuracy of laboratory value extraction was evaluated on F1 (the harmonic mean of positive predictive value and sensitivity), on a held-out test set against ground truths provided by a team of clinicians. These values were reported with 95% CIs computed using the percentile bootstrap method with 1000 replicates.

## Results

### Association of AI Optimization With Information Extraction Time

**Figure 4.**
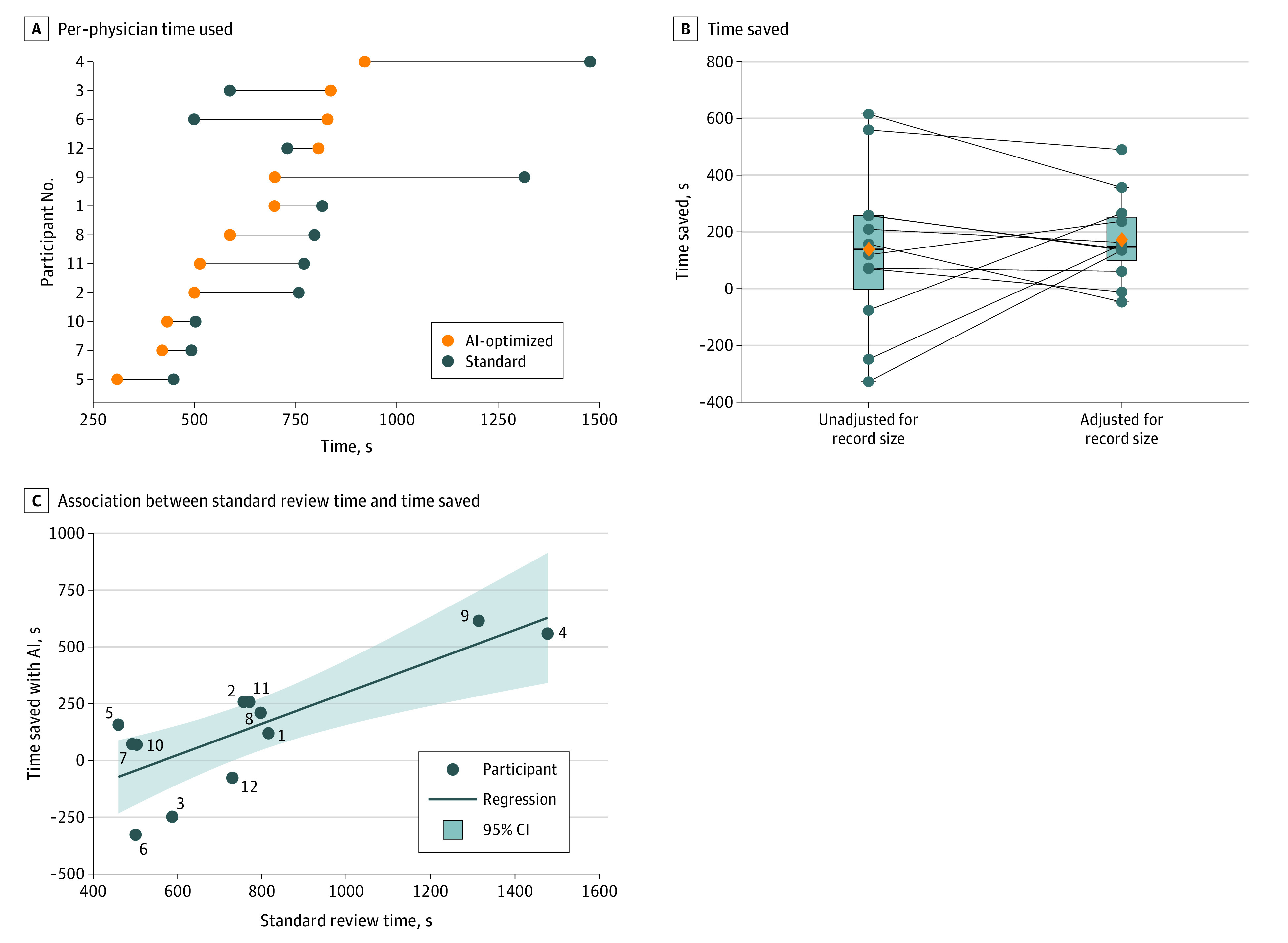
Time Saved by Artificial Intelligence (AI) Optimization A, Per-physician time taken for completion of questions for AI-optimized and standard review. Bars with an orange dot on the left and a blue dot on the right represent time saved with AI optimization; bars with a blue dot on the left and an orange dot on the right represent time lost. B, Time saved, adjusted for record size. The left side shows each physician’s time saved using AI-optimized review; the right side shows their time saved after using the time savings mixed model to standardize with a record size of 34 pages. Boxes represent quartiles (Q3-Q1), horizontal bars represent the median, and orange diamonds represent the mean. Error bars indicate the maximum and minimum values that are not outliers. Blue circles represent the time saved for each individual person. C, Association between standard review time and time saved with AI optimization. There was a correlation between the time physicians take to complete a standard review and the time saved with AI optimization (*r* = 0.80; *P* = .002).

A total of 12 clinicians participated. Compared with standard patient record review, the AI system had a time savings of 18% for physicians to answer the 22 clinical questions (10.5 [95% CI, 8.5-12.6] vs 12.8 [95% CI, 9.4-16.2] minutes; *P* = .02) (eTables 7 and 8 in the Supplement). Figure 4A shows the crude individual, per-physician times taken to complete the questions with and without AI optimization. After standardizing our time-savings mixed model with a standard record size of 34 pages (mean size of the 4 patient records used for testing), we can see a reduction in the variation of time savings with the AI-optimized software (Figure 4B). The 3 individuals who did not have a crude savings from AI optimization would have saved time if packet size were standardized. Likewise, 2 individuals who saved time would no longer have had time savings associated with our AI system.

### Association of AI Optimization With Clinician Accuracy

The accuracy of answers to the standardized questions was comparable with and without AI optimization. Physicians using AI-optimized record extraction to answer the 22 questions demonstrated comparable accuracy compared with standard extraction (83.7% [95% CI, 79.3%-88.2%] with vs 86.0% [95% CI, 81.8%-90.2%] without AI-optimized records; *P* = .81) (eTable 9 in the Supplement).

### Association Between Standard Review Time and Time Saved for Physicians

In general, physicians who took longer to complete patient data extraction without AI optimization saved the most time from AI-optimized referral records. There was a linear association between these variables (*r* = 0.80; *P* = .002) (Figure 4C).

### Performance of Software

The 4 records used for evaluation consisted of 136 pages of medical records. Of these, 119 pages were correctly classified for date, with an overall accuracy of 87.5% (95% CI, 80.9%-92.0%), and 109 pages were correctly classified into the right category, with an overall accuracy of 74.3% (95% CI, 66.9%-81.7%). By contrast, a majority-class baseline, where the most common class in the data set is always predicted (“note” in this case), achieved an accuracy of 50.0% (95% CI, 41.1%-58.8%). When evaluated on laboratory name extraction only, the laboratory extraction system achieved an F1 of 88.0% (95% CI, 82.35%-93.13%); when evaluated on both name and value extraction, the system achieved an F1 of 77.2% (95% CI, 67.9%-85.3%). Individual precision and recall and F1 metrics for each system are shown in eTables 4 to 6 in the Supplement.

### Physician Thoughts on AI and Performance

**Figure 5.**
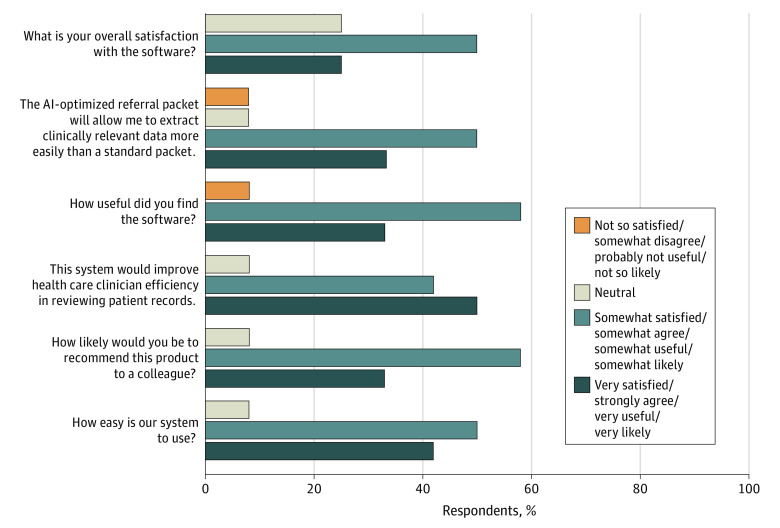
Subjective Feedback Results After the 2 evaluation referral records, a subjective feedback survey was administered. Feedback from clinicians on software utility and performance on a 5-point Likert scale was generally positive. For any given survey question or statement, the absence of a percentage of respondents indicates that there were no responses for that category. AI indicates artificial intelligence.

Most of the feedback from clinicians on software utility and performance was positive (Figure 5). Eleven of 12 clinicians (92%) found the software useful and agreed, to various extents, that it would improve clinician efficiency in reviewing patient records. Clinicians reported that the software could provide an estimated time savings ranging from 5 to 30 minutes; the mean (SD) savings estimated was 14.5 (11.1) minutes when reviewing a new patient record. Most physicians (11 of 12 [92%]) reported that they preferred the AI-optimized record and would be interested in using it in their clinic. The full results from our user experience survey are presented in eTable 10 in the Supplement.

## Discussion

The purpose of this study was to design and evaluate an AI-based system to assist physicians in extracting information from patient referral records. Our approach incorporated both visual and text information of each page of a record to identify important clinical information, including relevant dates, page content, and laboratory values. In addition, this information was presented in a user interface that displayed the extracted information in a convenient manner alongside the original record, allowing physicians to review patient referral records quickly and easily.

Previous work in clinical information harvesting has largely focused on extraction from structured information found in electronic health records, such as laboratory values and imaging results.^19,21,22^ However, referral records are heterogenous with multiple types of data and without common inherent structure and thus require more complex methods. Some prior work has applied natural language processing techniques to extract information from unstructured, free-form text, but these systems were not evaluated in a setting simulating clinical workflow.^25,26^

To our knowledge, this work is the first to use a novel AI system to facilitate the review of patient referral records, a key task in all medicine specialties. Unlike most previous work in text extraction, our system included a web-based user interface that was used to present clinical information to physicians. In addition, our system processed raw, scanned referral records, a more challenging problem that more accurately represents the clinical scenario physicians deal with regularly in working with various forms of unstructured data. Although 1 previous study has investigated spelling correction for medical OCR,^27^ it did not use the extracted information for additional downstream tasks such as answering clinical questions.

Our study demonstrated a mean time savings of 2.3 minutes (18% of standard review time) when clinicians used AI-optimized records to answer clinical questions, while achieving similar accuracy, compared with when AI optimization was not used. Notably, the 22-question quiz (and preceding training video and 3 test questions) was the first time physicians were exposed to the AI software. As with the adoption of many technologies, there may potentially be extra time savings had physicians had more experience with the user interface. We also showed that those who spent more time on data extraction using standard record review would benefit most from AI-optimized review. This is an important association because it can estimate which physicians may gain the most from the use of such an AI system in actual clinical practice.

The time difference required to answer the standardized questions with and without AI optimization was used as a surrogate marker of potential time savings in reviewing new patient referral data in actual practice. Many factors including referral packet size (mean size, 34 pages in our study) and data complexity would certainly be contributing factors in determining actual time savings using AI. Although it is difficult to accurately translate time savings of 18% in answering a set of standardized questions to time savings in real patient visits, clinicians reported that they thought this software could provide an estimated time savings of 14.5 minutes per new patient encounter. This potential time saved could be significant, considering that new patient encounters last at least 30 minutes at our and other institutions and may be used to increase time with existing patients or even open new patient visit slots to decrease wait times for subspeciality clinics.

Our results indicate a positive AI experience and desire by physicians to use such a system in their practice. Overall, 11 of 12 clinicians (92%) reported a preference for using the software compared with standard record review and would be interested in using this type of software in their clinics. The single clinician who expressed uncertainty about using the software had concerns regarding the amount of clicks it would take to go to various pages of the packet in our user interface and thought this could be an inconvenience. In general, the supportive responses of our survey highlight the importance of this issue as an area of need that can likely be generalized and expanded to multiple other medical subspecialties that share similar challenges, because many referral records contain similar types of information (eg, progress notes, radiology reports, pathology findings, procedure notes, etc).

### Limitations

Several important limitations of our study need to be acknowledged. Technical issues related to our machine learning model, including examples mentioned in clinician feedback, such as having a dedicated medication tab, a search function, and improved page classification can be viewed eTables 4 to 6 in the Supplement. This system, however, could readily be optimized further based on such feedback; future iterations of our initial system could also include more formal user-centered design processes. In addition, it is important to view our AI software in the context of our current EHR-based systems. Incorporation of an AI system such as ours into the EHR would be most useful to clinicians, because having to use a separate web-based user interface to access referral records may be an additional barrier to use. Furthermore, as barriers to health information exchange across currently disparate systems improve, clinicians will be able to more easily access information. However, we still believe there will be a need for technologies such as our AI system, because achieving data liquidity has typically been a gradual process. In addition, the need to better organize this information for extraction, even if aggregated, will remain and may actually become more pronounced as data volume increases.

As noted above, we do acknowledge the difficulties of applying our time savings in answering a set of standardized questions to extrapolate the time savings clinicians would have when reviewing a new patient record. Nonetheless, we believe our questions reflect the type of data that a clinician would need to consider when reviewing a new patient referral packet. In addition, although we have a relatively small number of participants (n = 12), they each answered numerous questions (44 total) and as such, we had adequate power to detect the nearly 20% time savings owing to AI optimization. With larger records and increased use of such an AI system, we hypothesize even more pronounced time savings. That said, we recognize that some users, particularly those who review existing referral packages rapidly, may have limited improved efficiency from the use of an AI system (Figure 4C).

Our classification accuracy could be enhanced and should be optimized in future iterations, despite our existing improvements over baseline being statistically significant. However, we note that the ontology of patient records is inherently somewhat ambiguous. For example, a clinical note largely detailing radiology results (and perhaps the only record including mention of such a radiology report) could reasonably be classified by a physician as either radiology report or a note. The physicians establishing the classification standard often preferred the note category, but our model often labeled such examples as radiology instead. Future work could treat this as a multilabel problem to avoid this ambiguity. In addition, this single center study comes with biases, especially when considering the subjective feedback responses of clinicians. Future studies at various sites and even potentially across different subspecialties could add additional strength to our findings.

## Conclusions

In this prognostic study, we designed an AI system to summarize and organize patient referral records for ease of clinician use. The AI system helped physicians extract relevant patient information in less time while maintaining high accuracy. This is particularly relevant in an era in which practitioners are confronting increasing volumes of EHR data and the loss of face-to-face interaction with patients.

## Notes

Supplement. **eMethods.** Procedures **eTable 1.** Keywords for Content Categorization **eTable 2.** Timed Clinical Questions **eTable 3.** User Experience Survey Questions **eTable 4.** Date and Content Categorization Accuracy per Packet **eTable 5.** Per-Class Content F1 **eTable 6.** Per-Packet Lab Extraction F1 **eTable 7.** Time Performance per Physician With and Without AI Optimization **eTable 8.** Time Performance per Question Across Physicians With and Without AI Optimization **eTable 9.** Accuracy Performance of Physicians With and Without AI Optimization **eTable 10.** Survey Outcomes Counts per Answer
