# Supplementary material for: Development and Validation of an Artificial Intelligence System to Optimize Clinician Review of Patient Records
Source: JAMA Netw Open. 2021 Jul 23;4(7):e2117391. doi: 10.1001/jamanetworkopen.2021.17391 (PMC8303101; doi:10.1001/jamanetworkopen.2021.17391)
Supplement: Supplement. — eMethods. Procedures eTable 1. Keywords for Content Categorization eTable 2. Timed Clinical Questions eTable 3. User Experience Survey Questions eTable 4. Date and Content Categorization Accuracy per Packet eTable 5. Per-Class Content F1 eTable 6. Per-Packet Lab Extraction F1 eTable 7. Time Performance per Physician With and Without AI Optimization eTable 8. Time Performance per Question Across Physicians With and Without AI Optimization eTable 9. Accuracy Performance of Physicians With and Without AI Optimization eTable 10. Survey Outcomes Counts per Answer [file jamanetwopen-e2117391-s001.pdf]

## Supplementary Online Content

Chi EA, Chi G, Tsui CT, et al. Development and validation of an artificial intelligence system to optimize clinician review of patient records. *JAMA Netw Open*. 2021;4(7):e2117391. doi:10.1001/jamanetworkopen.2021.17391

**eMethods.** Procedures
**eTable 1.** Keywords for Content Categorization
**eTable 2.** Timed Clinical Questions
**eTable 3.** User Experience Survey Questions
**eTable 4.** Date and Content Categorization Accuracy per Packet
**eTable 5.** Per-Class Content F1
**eTable 6.** Per-Packet Lab Extraction F1
**eTable 7.** Time Performance per Physician With and Without AI Optimization
**eTable 8.** Time Performance per Question Across Physicians With and Without AI Optimization
**eTable 9.** Accuracy Performance of Physicians With and Without AI Optimization
**eTable 10.** Survey Outcomes Counts per Answer

This supplementary material has been provided by the authors to give readers additional information about their work.

© 2021 Chi EA et al. *JAMA Network Open*.

## eMethods. Study Procedures

First, a date extraction algorithm was developed to identify the date from a group of pages in a referral record; we defined this as the most important date for a given particular group (typically the encounter or result date). Each individual page within the group was searched for dates. In order to exclude dates that were irrelevant to a patient's visit, only dates within the body of each page were considered, with those in the header or footer excluded. Additionally, dates before the year 2000 or in the future were ignored, as they were assumed to be incorrectly parsed or of marginal clinical utility. After this filtering, the most recent of the remaining dates was outputted as the predicted date for a given group. Second, a lab extraction algorithm to extract a list of lab values from a referral record containing such data was developed. The entire referral record was searched for lab names, such as creatinine or hemoglobin, as well as common abbreviations. The extracted lab values were organized into a table sorted by lab name and date.

To organize the record by content category, first, we developed a content categorization algorithm to classify the type of information in a body of text into one of the following categories: Referral, Note, Lab, Radiology, Procedure, Operative Report, Pathology, Fax Cover Sheet, or Insurance. Guided by physician input, a set of keywords that would best discriminate between these categories was developed by reviewing patient records (eTable 1). Input text was searched for keywords in the set, then classified as the category which best matched the keywords found. Subsequently, we developed a page grouping algorithm to use both visual and textual information to partition a referral record into its constituent documents. To do so, a convolutional neural network was developed to take in an image of a page as input and then predict whether the page was the first page of a document. The neural network's prediction was then combined with textual heuristics, such as the presence of phrases of the form "Page X of Y," as well as information from the content categorization algorithm, to produce a final prediction. Based on these predictions, an input record was partitioned into its constituent documents, with each predicted first page beginning a new document. This partitioning was used to perform date extraction and content categorization at the document level, rather than the page level. This led to greater accuracy for both tasks, since textual information from all the pages in a document could be combined for prediction.

To present the optimized patient information to the clinician, we developed a web interface that displayed the outputs of the system for a given referral record. Displayed on the left side of the interface was a summary containing a list of document categories found in the record, along with hyperlinks to the original full PDF record, which was shown on the right side of the interface (Figure 2). Each category on the left displayed a list of nested items predicted by the system to belong to that category, along with the date of that document. For example, in the radiology category, all CT scans, MRIs, and other radiographic pages would be listed with their dates of completion. Additionally, lab values extracted from the document were displayed in a table organized by date. Social information such as smoking history and allergies were displayed in a separate section. All categorized documents were linked to the original PDF: clicking on any document on the summarized display section on the left of the interface would scroll to the page in the original full PDF record on the right side of the interface from which it was extracted.

We developed a system to extract relevant clinical information from patient referral records. A referral record consists of many scanned documents, generally concatenated together into one file. First, optical character recognition (OCR) was applied to parse the PDF into text. The pages were then split into *groups*—sets of consecutive pages that make up a single source document. To do so, a convolutional neural network, augmented by a set of textual heuristics, was applied to each page to predict which pages began a new document. For each group, manual heuristics were applied to extract the date, and a manually tuned linear classifier was applied to predict the document category. Position- and text-based

© 2021 Chi EA et al. *JAMA Network Open*.

heuristics were run over the entire lab record to extract a list of lab values, as well as information regarding smoking and allergies.

**Text Extraction:** Extraction of text from referral records was done with PyOCR (0.7.2), an optical character recognition library using the Tesseract 4.1.1 engine. We chose PyOCR for its flexibility in reading all image types, its various output types for text, and its ability to parse only digits within a text, a feature essential in the extraction of specific lab values. Every page was converted first to an image, before the algorithm was used to detect all regions of text on the page. The algorithm returned a list of results; each result contained the coordinates of the bounding rectangular box as well as the text contents within it. We used the PyOCR library, an optical character recognition (OCR) tool wrapper that uses the Long Short-Term Memory-based Tesseract 4.1.1 OCR Engine.

**Date Extraction:** The date extraction component determines the timestamp of a group of pages. On each page, a set of keywords (eTable 1) were used to select a set of text boxes to be searched for timestamps. To avoid date artifacts from the times faxes were received, all dates with a bounding box with the vertical coordinate of its left top corner positioned on the top or bottom 10% of the page were discarded. All remaining parseable dates were extracted, and the most recent remaining date more recent than the year 2000 was selected.

**Lab Extraction:** The lab extraction component uses a list of lab names along with their corresponding numerical ranges to extract a list of lab values from the lab tables in a patient referral record. First, the referral record was searched for lab names or their common abbreviations. Common OCR misreadings were also included in the list of abbreviations. For each instance of a lab name that was found, the area to the right of the lab name on the page was searched for numerical values. Values that fell outside a clinically specified range were excluded; the first remaining numerical value on the line was selected.

**Content Categorization:** The content categorization component categorizes a given page or group of pages as one of a set of predetermined categories: Referral, Note, Lab, Radiology, Procedure, Operative Report, Pathology, Fax, and Insurance. For each category, a characteristic set of keywords, along with a set of weights indicating how important each keyword is to classification, was developed in consultation with clinicians. When processing a referral record, each group of pages was searched for keywords; the total predicted score for each category was the sum of the weights for all keywords corresponding to that category found in the group. To minimize the impact of incidental mentions of information from other categories in long paragraphs, keywords found in a line at least 5 words long were weighted with a penalty of 0.3. For a given group of pages, the category with the highest predicted score was selected and displayed to the user.

The set of keywords and weights was developed alongside a clinician through an iterative procedure using the training records and a separate set of validation records. The clinician read through the documents in the training records and determined the category of each page. Then, for each class, they identified words contained in the training documents that were present in many of the documents of that class, and at the same time mostly absent from documents in other classes. Next, the list of words was fine-tuned on the validation set by testing how well the keywords separated pages into their correct classes when used with the content categorization procedure described above. The list was adjusted until the pages of the validation records were categorized at an acceptable level of accuracy.

**Document Partitioning:** The document partitioning component splits a referral record into its constituent documents. Referral records are typically made up of a large number of separate original documents

© 2021 Chi EA et al. *JAMA Network Open*.

merged into a single PDF; our algorithm recovers these documents by predicting whether each page is the beginning of a separate document. Each page of a referral record was first resized to 216x256 pixels, then converted to black and white. To classify a given page, the previous page, the current page, and the next page were input to a fine-tuned ResNet18 model pre-trained on ImageNet. The model was fine-tuned to classify pages on our training set of referral records for 15 epochs with a batch size of 64, using binary cross-entropy loss and the Adam optimizer with $\beta_1 = 0.9$, $\beta_2 = 0.999$, and a constant learning rate of 1e-4.

When processing a new referral record, each page was processed by our model; pages predicted to be a first page with probability greater than 0.5 were labeled as the beginning of a document. In addition, pages whose text contained "Page 1 of" were also classified as the beginning of a document.

**Web Implementation:** We developed a web interface for clinicians that organized the output of the system for a given patient record. A main *group view* was organized into sections corresponding to document category. Each section displayed a list of page groups that were predicted to belong to that category; additionally, each group was rendered with a differently-colored label to better distinguish between categories. Within each section, the groups were sorted in original page order and labeled with the predicted date. Any duplicate groups were rendered in grey below their duplicate (Figure K). A *lab view* displayed a table of labs found in the patient record. Rows corresponded to a given lab type (e.g. calcium); columns corresponded to a specific date. A *social history view* displayed pages predicted to contain information about allergies and social history. Our interface consisted of a split-screen layout, with the summarized outputs of our algorithm on the left and the original PDF record on the right for cross-reference. Dragging the border between the two screens allowed for resizing the two panes. Clicking on a link in the summary (i.e., a page group, a lab value, or a social history link) scrolled to the appropriate page of the PDF record. Conversely, scrolling in the PDF record scrolled the summary to the appropriate section. A screenshot of the interface is presented in Figure 2. We implemented our system using the jQuery library (version 3.4.1) and pdf.js (version 2.3.2).

**Selection of patient records/categories for referral packet organization:** We randomly chose new patient referral packets from a variety of gastroenterology providers at Stanford. They are typical of a wide variety of gastroenterology referrals as they were taken across multiple sub-specialties including general GI, liver disease, GI motility, etc.

Our AI system includes all pages of the original referral packet, but predicts and categorizes each page of the packet into of nine categories that are most commonly present in a typical referral packet. The rationale for these nine are that patient notes, labs, radiology/operative/procedure/pathology reports are useful patient information and referral page, cover sheet and insurance information is often included in these referral packets as well. In consultation with physicians in our research group, we determined that the most useful parts of extraction would be dates, in order to chronologically order notes as well as objective data (procedures and labs). In addition, a tabular format for routine labs was thought to be very helpful as it could provide insight into trends over time, which is often monitored in clinical practice.

**Limitations**

Our system has several technical limitations. First, when multiple dates were included in a given group and recognized by our date extraction algorithm, the most recent date was selected. However, this may not always correspond to the most clinically relevant date; for example, the day a lab procedure was performed may be more clinically important than the day the results were reported. In addition, our

© 2021 Chi EA et al. *JAMA Network Open*.

system currently displays all pages in the summary, even those with little information or with significant informational overlap with other pages. Although removing such pages would improve the ease of use of our system, such a modification could lead to the potential omission of important patient information, especially information incorrectly parsed by our OCR component. This could, however, be customized in a future version of this system. Additionally, our current duplicate-removal system, based solely on word-level overlap, is insufficiently sophisticated to identify pages with high semantic overlap. Future work might extend this with neural-based duplication detection.

**Mixed Effects Model**

```
proc mixed data=work;
class qid sid;
model time = assisted acc packetlength /solution cl;
repeated / subject=qid(sid);
```

This study was performed with a multi-reader multi-case (MRMC) strategy. To account for variabilities among readers and cases, a linear mixed effects regression model is used with random reader-specific and case-specific effects. Two random reader-specific effects and two random case-specific effects terms are included in the regression model.

$$Reading\_Time = b_0 + b_{0i} + b_{0j} + (b_{int} + b_{int\_i} + b_{int\_j})X_{int} + \varepsilon_{ij}$$

where:

$b_{0i}$ = reader-level random intercept assumed to follow a Gaussian distribution

$b_{0j}$ = case-level random intercept assumed to follow a Gaussian distribution

$b_{int\_i}$ = reader-level random slope assumed to follow a Gaussian distribution

$b_{int\_j}$ = case-level random slope assumed to follow a Gaussian distribution

$X_{int}$ = AI-optimization (Standard Review = 0, AI-optimization = 1)

$\varepsilon_{ij}$ = Gaussian error

Therefore, from the above model, both overall and reader-specific differences between the two groups are able to be estimated.

© 2021 Chi EA et al. *JAMA Network Open*.

**eTable 1. Keywords for Content Categorization**

***Caption:*** *A keyword followed by a number in brackets indicates its weight for linear weighting. If no number is listed, the weight is 1.*

| **Category** | **Keywords** (weight) |
|---|---|
| **Referral** | dear, sincerely, referral, insurance information, patient insurance, reason for referral, referral request, referring provider, referral form, outside provider, clinician request, patient information, documentation required, referral order, refer to, referred from, referred to |
| **Fax** | fax cover sheet, sender's information, recipients information, fax number, confidentiality notice, fax sheet, subject, fax from, company, department, fax to, cover page, authorisation, medical record |
| **Insurance** | insurance information, primary insurance, subscriber name, subscriber id, employer information, employer name, emergency contact name, health insurance card, primary health insurance, member, id, insurance, insurance details, subscriber, insurance company, policy holder, policy number, carrier, member number, member name, deductible, identification number, health plan, group name, plan sponsor, insurance type |
| **Note** | present illness, past history, family history, social history, surgical history, medications, review of systems, vital signs, physical exam, assessment/plan, medical history, vitals, patient encounters, summary/plan, assessments, reason for appointment, reason for visit, current symptoms, current meds, h&p, patient encounter, home meds, plan order summary, plan order, progress note, discharge record, dc record, order information, discharge summary, pleasure, ethnicity (10) |
| **Procedure** | procedure (0.2), procedure performed, estimated blood loss, indications, impression, recommendation, description of procedure, colonoscopy report, endoscopy report, colonoscopies polypectomy report, sigmoidoscopy report, procedure date, upper gi endoscopy, endoscopy, add'l images, additional images, procedure note, colonoscopy with (10), date of procedure, primary surgeon, assistant surgeon, description of procedure, postoperative, description of procedure, "procedure: colonoscopy", debris |
| **Radiology** | procedure (0.2), imaging services report, imaging report, small bowel , technique, fluoroscopy, ultrasound report, ultrasonography, pelvis, ct abdomen, with contrast, without contrast, enterography, indications, x-ray, MRI, MRE, imaging result, impression, imaging information, ultrasound, grayscale, doppler, doppler imaging, sonographic, enterography, enhanced, enterography, views, radiologist, tomography, views, radiology consultation (2), series, acute series |

© 2021 Chi EA et al. *JAMA Network Open*.

| | |
|---|---|
| **Lab***<br>(all keywords have weight 0.3 by default) | lab result report, lab report, serology results, genetic results, inflammation results, IgG, IgA, lab ref id, performing lab, ordered by, laboratory report, laboratory results report, rbc, abc, abc, haemoglobin, units, platelets, lab test report, laboratory test report, c-reactive protein, lab no, hematocrits, mcv, much, much, raw, platelet count, neutrophil, lymphocyte, monocyte, eosinophil, basophil, immature granulocyte, nucleated abc, creatinine, egfr, general chemistry, c reactive protein, albumin, libc, iron, glucose, sodium, potassium, chloride, calcium, gastroenterology, iron saturation, white blood cell, red blood cell, reference range, assay, amplification, laboratories, laborator, toxin, culture, salmonella |
| **Operative Reports** | procedure (0.2), timeouts, timeout details, timeout questions, pre-incision, patient preparation, closing documentation, supplies, operative report, primary surgeon, assistant surgeon, anaesthesiologist, preoperative, postoperative, description of procedure, complications, cross-clamp, estimated blood loss, transplant, bypass, choledochocholedochstomy, ectomy, ligament, cannulate, anastomose |
| **Pathology** | biopsy report, pathology report, biopsy, specimen (0.2), histopathology, microscopic diagnosis, gross description, surgical pathology, pathology laboratory, microscopic findings, diagnosis (microscopic), microscopic description, specimen submitted, gross tissue description |

© 2021 Chi EA et al. *JAMA Network Open*.

**eTable 2. Timed Clinical Questions**

***Caption**: Listed are the questions given to physicians to answer for each standard or AI-optimized packet. All questions were timed; only one question appeared on the interface at a time, and had to be submitted before the next question could be viewed.*

| # | Question | Options |
|---|---|---|
| 1 | How many endoscopies (upper endoscopy or colonoscopy) has the patient received? | # |
| 2 | Is there an endoscopy pathology report? | *Yes/No* |
| 3 | Is there a report for a full diagnostic CT scan of the abdomen or pelvis? | *Yes/No* |
| 4 | What is the value of the patient's most recent Creatinine? | *Cr: __* |
| 5 | What is the date of the patient's most recent Creatinine? | *Date: ___* |
| 6 | What is the value of the patient's most recent Hemoglobin level? | *Hgb:___* |
| 7 | What is the date of the patient's most recent Hemoglobin level? | *Date: ___* |
| 8 | Is there a PPI listed on any medication list? | *Yes/No* |
| 9 | Does the patient actively smoke? | *Yes/No/No Data* |
| 10 | What was the date of the patient's last visit with a physician? | *Date: ___* |
| 11 | Does the patient have any allergies? | *Yes/No/No Data* |
| 12 | What is the patient's most recent GGT? | *Value: __ Date:___ / No Data* |
| 13 | Is there a radiology report for an ultrasound? | *Yes/No* |
| 14 | Is there a surgery operative report present? | *Yes/No* |
| 15 | Is there Aspirin, Ibuprofen or Naproxen listed in the medication list? | *Yes/No* |
| 16 | What is the value of the patient's most recent C-Reactive Protein (CRP)? | *Value: __ / No Data* |
| 17 | What is the date of the patient's most recent C-Reactive Protein (CRP)? | *Date: __ / No Data* |
| 18 | How many gastroenterology/hepatology office visit notes are in the chart? (For duplicate notes, only count the note once.) | # |
| 19 | When was the last gastroenterologist/hepatology office visit? | *Date:___ / No Data* |
| 20 | How many MRI of the abdomen or pelvis (including MRE) reports are present? | # |
| 21 | Does the patient have a history of bowel resection (excluding appendectomy) ? | *Yes/No* |
| 22 | Please list one of the patient's insurance providers. | |

© 2021 Chi EA et al. *JAMA Network Open*.

**eTable 3. User Experience Survey Questions**

***Caption:*** *Listed are the questions used in the experiment, given to physicians to answer after the completion of the quiz on an AI-assisted and standard packet. This survey was untimed, and allowed physicians to give in-depth feedback on our packet.*

| # | QUESTIONS |
|---|---|
| 1 | **What is your overall satisfaction with the software to optimize patient referral records?**<br>very satisfied – somewhat satisfied – neutral – not so satisfied – not at all satisfied<br>Any comments: |
| 2 | **Please rate your level of agreement with the following statement: The software-optimized referral record will allow me to extract clinically relevant data (information that you believe would affect patient care) more easily than a standard referral record.**<br>strongly agree – somewhat agree – neutral – somewhat disagree – strongly disagree |
| 3 | **How useful did you find the software?**<br>Very useful – somewhat useful – neutral – probably not useful – definitely not useful<br>**If you selected “not useful” what can we do to make it more useful?** |
| 4 | **Please select the aspects of the software which you find useful if any:**<br>Overall organization (categorization of notes, labs, procedures, etc.)<br>Lab table<br>Ability to link to original referral packet<br>Identification of duplicate data<br>Other: _______________ |
| 5 | **Please select which method you would prefer to review referral records:**<br>Software-optimized referral packet<br>Standard referral packet<br>No preference<br><br>**Any comments?** |
| 6 | **Please rate your level of agreement with the following statement: This software would improve health care provider efficiency in reviewing patient records.**<br>strongly agree – somewhat agree – neutral – somewhat disagree – strongly disagree |
| 7 | **Please estimate how much time, if any, on average the software may save you when reviewing a new patient record compared to standard record review:** ____ minutes |
| 8 | **Would you be interested in using this type of software in your clinic?**<br>Yes No Uncertain<br>**Any comments** |
| 9 | **How likely would you be to recommend this product to a colleague?**<br>very likely – somewhat likely – neutral – not so likely – not at all likely<br>**If you selected “not likely”, please tell us why:** |
| 10 | **How easy is the software to use?**<br>very easy – somewhat easy – neutral – not so easy – not at all easy<br>**If you selected “not easy”, please tell us why:** |
| 11 | **Please describe the feature(s) of our software that you found LEAST useful?** |
| 12 | **What is the most important feature we should add?** |

© 2021 Chi EA et al. *JAMA Network Open*.

| | |
|---|---|
| 13 | **Please offer your thoughts on how to improve this software or any issues you have with the software**. |

© 2021 Chi EA et al. *JAMA Network Open*.

**eTable 4. Date and Content Categorization Accuracy per Packet**

***Caption:*** *For each page within a packet, our algorithm output a date and class classification that was used to sort the packet into relevant groups. In this table, we evaluated the accuracy of these outputs, along with 95% CI's computed via the bootstrap replicate method, on each packet when compared to the ground truth provided by physicians. For dates, we evaluated both the percentage of pages in the packet labeled with the correct date, and the percentage labeled within 3 days of the correct date; both of these results are above 72% for all packets used in the experiment. The latter condition allows us to evaluate the accuracy of our model when ignoring small errors immaterial to clinical judgements. We achieve a similar, though slightly lesser, accuracy for class classification. The average is weighted so that each page has equal importance.*

| **Packet** | **Date Accuracy—24h [%] (95% CI)** | **5 Date Accuracy—72h [%] (95% CI)** | **Content Accuracy (95% CI)** | **# Pages** |
|---|---|---|---|---|
| 1 | 94.0 (87.4, 100.0) | 96.0 (90.6, 100.0) | 76.0 (64.2, 87.8) | 50 |
| 2 | 83.7 (72.7, 94.8) | 88.4 (78.8, 98.0) | 83.7 (72.7, 94.8) | 43 |
| 3 | 100 (100, 100) | 100 (100, 100) | 88.9 (74.4, 100) | 18 |
| 4 | 72.0 (54.4, 89.6) | 72.0 (54.4, 89.6) | 44.0 (24.0, 64.0) | 25 |
| **Average** | 87.5 (80.9, 92.0) | 89.7 (83.5, 93.8) | 74.3 (66.9, 81.7) | 34 |

© 2021 Chi EA et al. *JAMA Network Open*.

**eTable 5. Per-Class Content F1**

***Caption:*** *We evaluated our content categorization algorithm per-packet. Four of the most common classes (Note, Radiology, Lab, and Procedure) achieved significant F1 scores > 0.7.* An asterisk (*) denotes that our result is significant at p<0.05.

| **Class** | **F1 (95% CI)** | **N** |
|---|---|---|
| Note | 0.738* (0.635, 0.828) | 68 |
| Radiology | 0.857* (0.741, 0.943) | 32 |
| Lab | 0.816* (0.681, 0.913) | 8 |
| Procedure | 0.777* (0.500, 0.952) | 5 |
| Pathology | 0.400 (0.000, 1.000) | 5 |
| Undetermined | 0.182 (0.000, 0.545) | 4 |
| Referral | 0.429 (0.000, 0.800) | 3 |
| Insurance | 0.666 (0.000, 1.000) | 3 |
| Fax | 0.750 (0.000, 1.000) | 2 |

© 2021 Chi EA et al. *JAMA Network Open*.

**eTable 6. Per-Packet Lab Extraction F1**

***Caption:*** *For each packet, our lab extraction algorithm identifies all pages including lab values and extracts the relevant fields to put in an aggregate table. We evaluated our algorithm's F1 recall score on both extracting lab names and extracting both the lab name and value.*

| **Packet** | **Name only (95% CI)** | **Name + Value (95% CI)** |
|---|---|---|
| 1 | 0.9500 (0.8649, 1.000) | 0.7647 (0.5517, 0.8947) |
| 2 | 0.9286 (0.8000, 1.000) | 0.8462 (0.6957, 0.9655) |
| 3 | 0.7500 (0.5714, 0.8889) | 0.7273 (0.4444, 0.8800) |
| 4 | 0.8235 (0.6667, 0.9474) | 0.7500 (0.5714, 0.8889) |
| **Combined** | **0.8800 (0.8136, 0.9313)** | **0.7719 (0.6792, 0.8525)** |

**Mapping:** {Subject51: Participant 1, Subject52: Participant 2, Subject54: Participant 3, Subject55: Participant 4, Subject57: Participant 5, Subject58: Participant 6, Subject59: Participant 7, Subject60: Participant 8, Subject61: Participant 9, Subject62: Participant 10, Subject64: Participant 11, Subject67: Participant 12}

© 2021 Chi EA et al. *JAMA Network Open*.

**eTable 7. Time Performance per Physician With and Without AI Optimization**

***Caption:*** *Note that each assisted time and unassisted time are not directly comparable per-physician due to each physician taking two different packets.*

| | AI-Optimized Review Time [sec] | Standard Review Time [sec] |
|---|---|---|
| **Participant 1** | 697 | 816 |
| **Participant 2** | 500 | 757 |
| **Participant 3** | 837 | 588 |
| **Participant 4** | 919 | 1478 |
| **Participant 5** | 303 | 460 |
| **Participant 6** | 828 | 500 |
| **Participant 7** | 420 | 492 |
| **Participant 8** | 588 | 797 |
| **Participant 9** | 699 | 1314 |
| **Participant 10** | 433 | 503 |
| **Participant 11** | 513 | 771 |
| **Participant 12** | 806 | 730 |
| **Average** | 628.6 (95% CI: 517, 730) | 767.2 (95% CI: 584, 950) |

© 2021 Chi EA et al. *JAMA Network Open*.

**eTable 8. Time Performance per Question Across Physicians With and Without AI Optimization**
***Caption:*** *A mixed-effects analysis (alpha=0.05) was used to calculate the time benefit of AI-optimization per question. On average, our system provides an advantage of* ***8.09*** *seconds per question (****bold****).*

| Effect | Estimate | Std. Err. | DF | t Value | Pr > \|t\| | Alpha | Lower | Upper |
|---|---|---|---|---|---|---|---|---|
| **Intercept** | 23.296 | 6.5195 | 227 | 3.57 | 0.0004 | 0.05 | 10.4495 | 36.1425 |
| **Assisted** | -8.0943 | 3.3816 | 225 | -2.39 | 0.0175 | 0.05 | -14.758 | -1.4305 |
| **Acc** | 1.7279 | 4.9912 | 225 | 0.35 | 0.7295 | 0.05 | -8.1076 | 11.5633 |
| **Packet Length** | 0.466 | 0.1275 | 225 | 3.65 | 0.0003 | 0.05 | 0.2147 | 0.7174 |

© 2021 Chi EA et al. *JAMA Network Open*.

**eTable 9. Accuracy Performance of Physicians With and Without AI Optimization**

***Caption:*** *Note that each assisted time and unassisted time are not directly comparable per-physician due to each physician taking two different packets.*

| | Assisted Accuracy [%] | Unassisted Accuracy [%] |
|---|---|---|
| **Participant 1** | 77.2 | 86.4 |
| **Participant 2** | 90.9 | 72.7 |
| **Participant 3** | 72.7 | 90.9 |
| **Participant 4** | 95.5 | 86.4 |
| **Participant 5** | 72.7 | 95.5 |
| **Participant 6** | 68.2 | 72.7 |
| **Participant 7** | 77.2 | 86.4 |
| **Participant 8** | 90.9 | 100 |
| **Participant 9** | 86.4 | 100 |
| **Participant 10** | 86.4 | 86.4 |
| **Participant 11** | 86.4 | 68.2 |
| **Participant 12** | 100 | 86.4 |
| **Average** | 83.7 (95% CI: 81.0, 86.4) | 86.0 (95% CI: 83.2, 88.8) |

© 2021 Chi EA et al. *JAMA Network Open*.

**eTable 10. Survey Outcomes Counts per Answer**

***Caption:*** *The effectiveness and practical utility of our system was evaluated through a post-quiz feedback survey; below are the complete results for categorical questions (n=12).* ***Bold*** *= most common response.*

| # | Response (1-5) | 1 | 2 | 3 | 4 | 5 |
|---|---|---|---|---|---|---|
| 1 | What is your overall satisfaction with the software to optimize patient referral packets? *(1 = very unsatisfied, 2 = not so satisfied, 3 = neutral, 4= somewhat satisfied, 5 = very satisfied)* | 0 | 0 | 3 | **6** | 3 |
| 2 | Level of agreement: The software-optimized referral packet will allow me to extract relevant data more easily. *(1 = strongly disagree, 2 = somewhat disagree, 3 = neutral, 4 = somewhat agree, 5 = strongly agree)* | 0 | 1 | 1 | **6** | 4 |
| 3 | How useful did you find the software? *(1 = not useful, 2 = probably not useful, 3 = neutral, 4 = somewhat useful 5 = very useful)* | 0 | 1 | 0 | **7** | 4 |
| 5 | Level of agreement: This software would improve health care provider efficiency in reviewing patient records. *(1 = strongly disagree, 2 = somewhat disagree, 3 = neutral, 4 = somewhat agree, 5 = strongly agree)* | 0 | 0 | 1 | 5 | **6** |
| 8 | How likely would you be to recommend this product to a colleague? *(1 = very unlikely, 2 = not so likely, 3 = neutral, 4 = somewhat likely, 5 = very likely)* | 0 | 0 | 1 | **7** | 4 |
| 9 | How easy is our software to use? *(1 = very difficult, 2 = somewhat difficult, 3 = neutral, 4 = somewhat easy, 5 = very easy)* | 0 | 0 | 1 | **6** | 5 |
| | **Response (options: Software-optimized referral packet/ Standard referral packet/ No preference)** | **Standard** | | | **Software** | |
| 4 | Please select which method you would prefer to review referral packets. | 1 | | | 11 | |
| | **Response (yes/no/uncertain)** | **Uncertain** | | | **Yes** | |
| 7 | Would you be interested in using this type of software in your clinic? | 1 | | | 11 | |
| | **Response (min)** | **5** | **10** | **15** | **20** | **30** |
| 6 | Please estimate how much time, if any, on average the software may save you when reviewing a new patient record.<br>* *note: one invalid response* | 5 | 1 | 1 | 1 | 3 |

© 2021 Chi EA et al. *JAMA Network Open*.
